# Is it Always Safe to Administer RAS Inhibitors to Patients Who Underwent Unilateral Radical Nephrectomy?

**DOI:** 10.1155/crin/5481521

**Published:** 2026-02-23

**Authors:** Giulio Romano, Alessandro Crestani, Giuseppe Como, Erika Cucchiaro, Nicholas Fiorini, Gianluca Colussi

**Affiliations:** ^1^ Nephrology Division, Department of Medicine, University of Udine, Udine, Italy, uniud.it; ^2^ Department of Medicine, Urology Unit, University of Udine, Udine, Italy, uniud.it; ^3^ Department of Medicine, Insitute of Radiology, University of Udine, Udine, Italy, uniud.it; ^4^ Division of Internal Medicine, Department of Medical Sciences, University of Ferrara, Ferrara, Italy, unife.it

## Abstract

Compensatory increases in single‐kidney glomerular filtration rate (GFR) are usually observed after unilateral radical nephrectomy. However, the influence of hemodynamically active therapies on early functional compensation remains uncertain. We describe a 65‐year‐old man with latent autoimmune diabetes of adulthood, nonproteinuric chronic kidney disease consistent with atherosclerotic nephropathy, and mildly reduced baseline renal function who underwent right radical nephrectomy with caval thrombus removal for clear cell renal cell carcinoma. Based on preoperative split renal function, a postnephrectomy GFR above 45 mL/min was anticipated. Despite continued treatment with ramipril and dapagliflozin, GFR at 3 months remained close to the immediate postoperative value. After discontinuation of both agents, GFR increased above the expected threshold and remained stable, without albuminuria. This observation is hypothesis‐generating and suggests that combined ACE and SGLT2 inhibition may transiently attenuate early functional compensation through hemodynamic mechanisms, particularly in patients with atherosclerotic nephropathy, supporting individualized perioperative management and close renal function monitoring.

## 1. Background

When performing a unilateral radical nephrectomy (URN), it is considered that the patient’s outcome will be favorable if the expected residual glomerular filtration rate (GFR), calculated as endogenous creatinine clearance, at 3 months postsurgery, is higher than 45 mL/min [1]. This prediction is based on a calculation in which the contribution of the residual kidney to the total GFR before nephrectomy is added as an estimated overfunction due to the so‐called “compensatory hypertrophy” (CH) of the residual kidney. On average, at 3 months, this increase represents about one‐fourth of the baseline renal function of the residual kidney before nephrectomy [2]. However, this estimate reflects an average population response and does not account for substantial interindividual variability related to age, vascular disease, and baseline nephron integrity. Therefore, the 45 mL/min threshold should be interpreted as a pragmatic prognostic marker rather than a strict physiological boundary.

The mechanisms causing CH after URN are poorly understood. Initial adaptations include enhanced renal plasma flow and a higher single‐nephron GFR (SNGFR), which are obtained via hemodynamic and growth‐factor mechanisms [3]. Unlike the hyperfiltration seen in proteinuric diabetic nephropathy [4], compensatory increases in SNGFR caused by nephron mass loss do not appear to be primarily dependent on systemic renin–angiotensin system (RAS) activation [5]. As a result, the continued use of angiotensin‐converting enzyme (ACE) inhibitors or angiotensin receptor blockers (ARBs) following nephrectomy has not consistently been associated with a failure of early functional compensation in the studied groups [6]. However, in vulnerable patients, ACE inhibitors/ARBs may reduce intraglomerular pressure via various mechanisms, potentially reducing the expected compensatory rise in SNGFR above the 45 mL/min safety threshold [7].

Nonetheless, information from current populations, including those taking sodium glucose cotransporter 2 (SGLT2) inhibitors, is limited, implying that patients receiving URN may require close monitoring and individualized care. Most foundational postnephrectomy compensation studies were conducted before SGLT2 inhibitors were widely used, limiting direct comparability to contemporary practice. While early functional compensation is typically favorable following nephrectomy, the long‐term consequences can vary among different patient phenotypes. Consequently, caution should be exercised when extrapolating findings across populations, particularly in the current context of dual ACE/ARBs and SGLT2 inhibitors use.

## 2. Case Presentation

We describe the case of a Caucasian male patient who, at the age of 60, was diagnosed with adult autoimmune latent diabetes (LADA). Antibodies against glutamic acid decarboxylase were positive, and treatment with insulin was started. Investigations into the possible complications of LADA revealed that he also suffered from nonproteinuric diabetic nephropathy and mildly reduced renal function, calculated as measured creatinine clearance (GFR 79.4 mL/min) [8]. Following this diagnosis, insulin therapy was started. From that moment on, the albumin‐to‐creatinine ratio (A/CR) was always less than 15 mg/g. Three years later, due to the onset of hypertension, therapy with ramipril was started (at a dosage of 5 mg/day), and GFR dropped from 71.8 to 61.3 mL/min.

**FIGURE 1:**
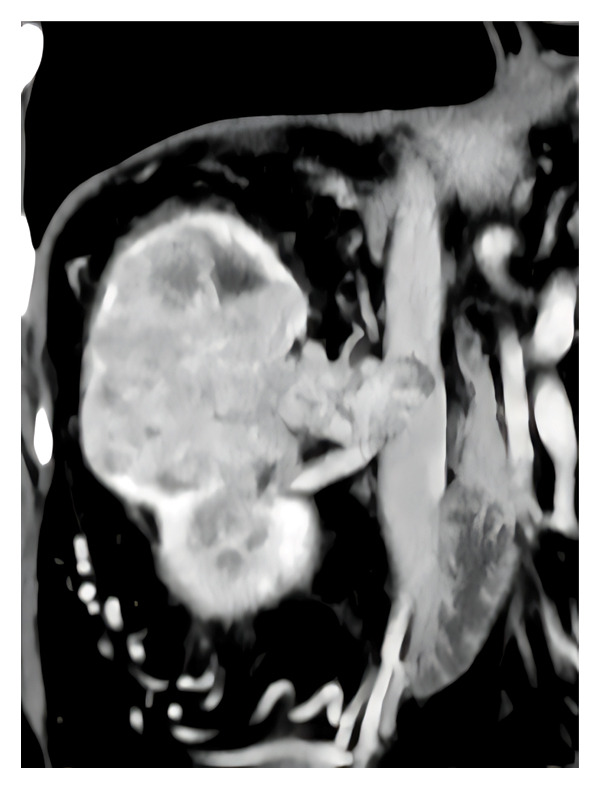
Contrast‐enhanced computed tomography (CT) scan of the abdomen showing a large right renal mass consistent with clear cell renal cell carcinoma, associated with extension into the inferior vena cava. The image illustrates the substantial tumor burden occupying the right renal fossa and its close anatomical relationship with the major vascular structures, supporting the indication for open right radical nephrectomy with caval thrombus removal. This preoperative imaging was also used for volumetric assessment of renal parenchyma to estimate split renal function prior to surgery.

To improve diabetes control, at the age of 64, the patient added dapagliflozin (10 mg/day), and GFR dropped further to 55.8 mL/min. An examination of the fundus oculi did not detect signs of diabetic retinopathy, but a renal Doppler ultrasound showed increased intraparenchymal renal resistive index (RRI), bilaterally equal to 0.72, without signs of renal artery stenosis. Nonproteinuric kidney disease associated with increased RRI suggested hypofiltering nephrons in the context of atherosclerotic nephropathy [9] rather than Kimmelstiel–Wilson’s glomerulonephritis [10]. At the age of 65 years, the patient received an incidental diagnosis of right clear cell renal cell carcinoma (Figure 1). He underwent open right radical nephrectomy with caval thrombus removal. Ramipril and dapagliflozin therapy were continued after nephrectomy, and blood pressure stabilized to the preoperative values after 2 days. No other major medication changes, intercurrent illness, or nephrotoxic exposures were documented during the perioperative period and the first three postoperative months.

To assess the contribution of the residual kidney to the total GFR before nephrectomy, in agreement with Rathi et al. [11], the prenephrectomy computed tomography (CT) scan was analyzed, which was performed to stage the disease (Table 1). In brief, an experienced radiologist manually performed slice‐by‐slice segmentation of both the kidneys and the tumor on contrast‐enhanced axial CT images using dedicated software (TeraRecon, Inc., 4000 E. 3rd Avenue, Suite 200, Foster City, CA 94404), followed by automatic interpolation and volume estimation (reported in cm^3^). The percentage contribution to the overall GFR of the residual kidneys was estimated at 80%. Therefore, given a prenephrectomy GFR of 55.8 mL/min, the residual kidney’s immediate postnephrectomy GFR would have been 44.6 mL/min. To this value, a further one‐fourth was supposed because of the CH after 3 months from nephrectomy, increasing the expected GFR to 55.3 mL/min, a value higher than the 45 mL/min threshold associated with an optimal nephrological patient’s outcome [1].

**TABLE 1:** Contrast‐enhanced computed tomography assessment (volume values expressed in cm^3^).

Prenephrectomy total renal parenchyma volume (excluding tumor)	254
Prenephrectomy left kidney volume (residual kidney)	204
Prenephrectomy right kidney volume	50
Tumor volume	300
GFR prenephrectomy (mL/min)	55.8
Contribution of the left kidney to the total filtrate (%)	80

**FIGURE 2:**
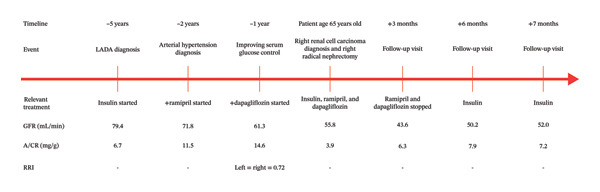
Changes in glomerular filtration rate (GFR), calculated as measured creatinine clearance, and albumin‐to‐creatinine ratio (A/CR) over time alongside key clinical milestones, including the initiation of ramipril, the addition of dapagliflozin, unilateral radical nephrectomy, and the discontinuation of both therapies. GFR at the time of nephrectomy was measured before the surgical intervention, and postoperative creatinine values were obtained in steady‐state conditions. This figure is designed to visualize temporal associations and does not imply causation. RRI: renal resistive index.

Unexpectedly, at the end of the 3 months, the patient’s GFR value, calculated as measured creatinine clearance, remained close to the immediate postoperative period measured in steady‐state conditions, standing at 43.6 mL/min. Following this, it was decided to discontinue the administration of both ramipril and dapagliflozin, and while the A/CR always remained less than 15 mg/g, the patient’s GFR started to rise up to 50.2 mL/min, a value well above the safety threshold of 45 mL/min. Renal function and A/CR remained stable, at 52.0 mL/min and 7.2 mg/g, respectively, after a further month of follow‐up. A single renin measurement obtained from an upright position following the discontinuation of ramipril and dapagliflozin was within the laboratory reference range (10 IU/μL; normal range: 3.5–46 IU/μL). This finding is reported descriptively due to the broad reference interval and the lack of serial endocrine and hemodynamic assessments, and it cannot be used to infer systemic or intrarenal RAS activity. Key laboratory values and treatment milestones are summarized in Figure 2 (clinical timeline).

## 3. Discussion

The surgical choice of radical nephrectomy in this patient was made in agreement with the guidelines of the American Urological Association [12] because of the inferior vena cava involvement and the risk of tumor relapse or dissemination associated with a nephron‐sparing surgery [13].

Following URN, an increase in single‐kidney filtration is common over the coming weeks to months, contributing to the expected improvement in global GFR. Despite an a priori prediction of functional compensation, this patient’s GFR at 3 months remained close to the early postoperative value and then increased after discontinuing both ramipril and dapagliflozin. This temporal association is consistent with the hypothesis that hemodynamic modulation of intraglomerular pressure may influence the early postoperative GFR trajectory in certain patients. Both ACE and SGLT2 inhibition are associated with reductions in intraglomerular pressure via distinct pathways, and combined exposure may plausibly contribute to blunting the short‐term functional component of compensation [14]. However, this report does not establish causality, and the observed GFR increase after drug withdrawal could be due to reversal of functional hemodynamic effects rather than structural adaptation. In addition, the discontinuation of both ramipril and dapagliflozin precludes any attribution to a single medication.

Multiple alternative explanations should be evaluated. Perioperative and early postoperative hemodynamics, such as transient hypotension, blood loss, volume shifts, and neurohormonal changes, can influence renal perfusion and filtration. However, granular intraoperative and early postoperative hemodynamic data were unavailable for a deeper analysis. The early GFR trajectory may be affected by drug‐related functional changes, such as the typically reversible GFR decrease associated with SGLT2 inhibitors [15], along with unmeasured intercurrent factors, including creatinine clearance variability, dietary intake, volume status, and other clinical changes. In addition, while no clinically recognized episodes of acute kidney injury were documented, the potential for subclinical perioperative renal injury persists. Renin, in particular, was assessed only once and without therapy; in the absence of serial measurements and supplementary data (e.g., aldosterone, sodium balance, and volume/hemodynamic profiling), a mechanistic interpretation of RAS activity status is impossible.

CH is primarily an adaptive response to the reduction of nephron mass, which supports short‐term functional reserves. It is important to recognize the heterogeneity and potential long‐term trade‐offs that may arise in vulnerable contexts, such as in atherosclerotic nephropathy. This patient presented increased RRI without significant albuminuria, which may suggest the coexistence of hypofiltering nephrons in the context of atherosclerotic nephropathy [16]. The primary clinical implication of this observation, especially in patients with suspected atherosclerotic nephropathy, is the need for careful monitoring of renal function and volume status during nephrectomy when hemodynamically active agents are used. This approach is preferred over the routine discontinuation of established cardiorenal protective therapy [17]. Consequently, we intentionally characterize CH as “adaptive” rather than “protective,” and we highlight monitoring‐based implications over mechanistic conclusions.

In humans who undergo donor nephrectomy, the adaptive increases in single‐kidney GFR are primarily due to hyperperfusion and structural adaptations. These increases can occur without showing sustained glomerular hypertension in many cases. Nevertheless, hyperfiltration may pose a long‐term risk, especially if kidney disease develops later, necessitating a phenotype‐specific interpretation. SGLT2 inhibitors help mitigate hyperfiltration through tubuloglomerular feedback mechanisms and typically lead to a transient, reversible dip in estimated GFR, followed by long‐term kidney protection. Consequently, early GFR trajectories postnephrectomy under dual therapy may indicate hemodynamic changes rather than a failure of structural compensation [18].

## 4. Conclusions

Within the limitations of a single case study, we observed that the expected early postnephrectomy increase in GFR was not evident at 3 months during combined treatment with ramipril and dapagliflozin, and GFR increased after discontinuation of both medications. This temporal association is hypothesis‐generating and does not establish a causal effect of ACE inhibition and/or SGLT2 inhibition on postnephrectomy functional compensation. Because perioperative hemodynamics, functional drug effects on intraglomerular pressure, and unmeasured confounders can influence early GFR trajectories, decisions regarding continuation or temporary adjustment of hemodynamically active therapies should be individualized and accompanied by close laboratory monitoring.

This consideration may be particularly relevant in patients with a predominantly vascular phenotype of chronic kidney disease, consistent with atherosclerotic nephropathy (e.g., low‐grade albuminuria with elevated intrarenal resistance indices), in whom GFR may be more sensitive to changes in renal perfusion and intraglomerular hemodynamics [16]. In such settings, therapies that lower intraglomerular pressure (ACEi/ARB and SGLT2 inhibitors) may be associated with a proportionally larger functional decline in GFR, particularly in the perioperative period, without necessarily implying structural kidney injury [14, 15]. Larger prospective studies are needed to determine whether specific subgroups are more susceptible to blunted early functional compensation following nephrectomy.

## Author Contributions

Giulio Romano and Nicholas Fiorini performed the background research and were the primary writers of the manuscript. Giulio Romano, Alessandro Crestani, and Erika Cucchiaro were involved in the clinical care of the patient. Giuseppe Como performed the interpretation of radiological images and provided high‐quality images for publication. Gianluca Colussi provided substantial revisions to the manuscript.

## Funding

No funding was received for this study. Open access publishing facilitated by Università degli Studi di Udine, as part of the Wiley ‐ CRUI‐CARE agreement.

## Disclosure

All authors have read and approved the manuscript.

## Ethics Statement

The authors have nothing to report.

## Consent

Written informed consent was obtained from the patient for publication of the case report including images and biochemical exams. A copy of the written consent is available for review by the editor of this journal.

## Conflicts of Interest

The authors declare no conflicts of interest.

## Data Availability

Data sharing is not applicable.
